# A T > G Mutation in the *NR5A2* Gene Is Associated With Litter Size in Hu Sheep Through Upregulation of Promoter Activity by Transcription Factor MTF-1

**DOI:** 10.3389/fgene.2019.01011

**Published:** 2019-10-25

**Authors:** Yinxia Li, Jun Zhang, Yong Qian, Chunhua Meng, Huili Wang, Jifeng Zhong, Shaoxian Cao

**Affiliations:** ^1^Institute of Animal Science, Jiangsu Academy of Agricultural Sciences, Nanjing, China; ^2^The Jiangsu Provincial Platform for Conservation and Utilization of Agricultural Germplasm, Jiangsu Academy of Agricultural Sciences, Nanjing, China

**Keywords:** Hu sheep, NR5A2, core promoter, single nucleotide polymorphism, competitive EMSA, MTF-1

## Abstract

Nuclear receptor subfamily 5 group A member 2 (NR5A2), also referred to as LRH-1 or FTF, is an orphan nuclear hormone receptor that is involved in regulating embryonic development, ovarian granulosa cell differentiation, gonadal sex differentiation, and steroidogenesis in mammals. However, little is known about how *NR5A2* regulates reproduction in sheep. In this study, we amplified the promoter sequence of *NR5A2* and determined that its core promoter region ranged from -721 nt to -281 nt. A T > G polymorphism at -700 nt was detected in the core promoter region. Association analysis found that the litter sizes of Hu ewes at their second and average parities with genotype GG (2.20 ± 0.20 and 1.97 ± 0.06, respectively) were significantly higher than those of ewes with genotype TG (1.68 ± 0.10 and 1.74 ± 0.05, respectively) (*p* < 0.05) and TT (1.67 ± 0.10 and 1.62 ± 0.06, respectively) (*p* < 0.05). The litter size of Hu ewes at their third parity with genotype GG (2.10 ± 0.10) was significantly higher than that of ewes with genotype TT (1.56 ± 0.12) (*p* < 0.05). A luciferase assay showed that the -700G allele increased the luciferase activity relative to the -700T allele. Furthermore, the -700T > G polymorphism created a novel binding site for metal-regulatory transcription factor 1 (MTF-1). A competitive electrophoretic mobility shift assay confirmed that MTF-1 specifically bound with the G-type promoter of *NR5A2*. An overexpression experiment demonstrated that MTF-1 was involved in the alteration of NR5A2 transcription activity and further increased *NR5A2* gene mRNA expression. Our findings revealed that the -700T > G polymorphism promoted *NR5A2* expression due to the positive effects on *NR5A2* gene transcription activity by MTF-1 and thereby increased fecundity in Hu sheep.

## Introduction

Nuclear receptor subfamily 5 group A member 2 (NR5A2), also referred to as liver receptor homolog-1 (LRH-1) or fetoprotein transcription factor (FTF) in *Drosophila* (Galarneau et al., 1996), is an orphan nuclear hormone receptor. It is widely expressed in all tissues, especially in the ovaries (Falender et al., 2003; Li et al., 2015), and is involved in embryonic development (Wei et al., 2018), ovarian granulosa cell differentiation (Meinsohn et al., 2017), gonadal sex differentiation (Yan et al., 2018), and steroidogenesis (Camats et al., 2015; Xiao et al., 2018).

Meinsohn et al. (2017) found that NR5A2 played a role in granulosa proliferation and follicle growth. The fecundity of heterozygous mice (NR5A2+/-) significantly decreased (Labelle-Dumais et al., 2007). Mice lacking NR5A2 in granulosa cells were sterile as a result of anovulation (Duggavathi et al., 2008). Kisspeptin neuron-specific NR5A2 knockout mice were shown to have significantly reduced litter sizes (Atkin et al., 2013). Polymorphisms in the *NR5A2* gene were associated with preterm birth in humans (Kaluarachchi et al., 2016) and litter size in Hu sheep (Li et al., 2015). The mRNA level of the *NR5A2* gene is significantly associated with the ovulation rate and litter size of Hu sheep (Li et al., 2015). This suggests that the *NR5A2* gene plays an important role in regulating the reproductive performance of female animals.

Our previous study demonstrated that polymorphism in the promoter region of the ovine *NR5A1* gene, another member of the NR5A family, is associated with litter size (Li et al., 2018). However, the polymorphism of the *NR5A2* promoter region and its relationship with litter size in Hu sheep is still unknown. In this study, we examined the 5′ regulatory region of the *NR5A2* gene, identified its core promoter region, screened SNPs in the core promoter region, determined the relationship of this gene with litter size in Hu sheep, and further explored the potential molecular mechanism of the *NR5A2* gene. All these data will be important in elucidating the molecular characteristics of NR5A2, understanding its function in regulating the litter size in Hu sheep, and comprehensively exploring the molecular mechanism of the NR5A family that is involved in regulating the high fecundity of sheep.

## Materials and Methods

### Samples

Ear tissue was collected from 193 Hu ewes (they are independent individuals and there is no relationship among them) from Helen Sheep Industry Co., Ltd., Jiangsu Province, China. DNA was extracted using a standard phenol–chloroform extraction protocol (Psifidi et al., 2010). Five healthy Hu ewes were chosen from the Dongshan Hu sheep conservation field in Jiangsu Province. After euthanization, eight tissue samples were collected (ovary, uterus, heart, liver, spleen, kidney, lung, and muscle tissue) and immediately placed in liquid nitrogen before being stored at -80°C. RNA was extracted from tissue samples using RNA extract kits (Bioteke, China). All experiments were performed in accordance with the protocol approved by the Committee on Ethics of Animal Experimentation from the Jiangsu Academy of Agricultural Sciences (No. 63 of Jiangsu Academy of Agricultural Sciences, approved on 8 July 2014).

### Cloning, Sequencing, and Genotyping

A PCR was conducted using a 20-μl mixture containing 1 μl of reverse transcription (RT) product or 60 ng of DNA, 2 U of Taq DNA polymerase (Takara, Japan), 2 μl of 10× PCR buffer, 0.5 mmol/L dNTP, 2.5 mmol/L MgCl_2_, and 0.5 μl of 10 nmol/L upstream and downstream primers (**Table 1**). Amplification conditions were as follows: 94°C for 4 min; 94°C for 30 s, annealing temperature (listed in **Table 1**) for 30 s, and 72°C for 30 s for 35 cycles; and 72°C for 5 min. DNA purification and sequencing methods followed those of Li et al. (2015).

**Table 1:** Primers used in this study.

primers	Primers sequence(5’-3’)	Tm(°C)	Size(bp)	Usage
P1	F: ATAGCTAGCCCATGCCTCTGATTAGAA R: ATACTCGAGGGCAGTCCTTTGGTTTAG	55	265	constructed pGL3-281 vector of *NR5A2* gene
P2	F: ATAGCTAGCACAAGAAGAGACGAACGG R: ATACTCGAGGGCAGTCCTTTGGTTTAG	56	705	constructed pGL3-721 vector and pGL3-G of *NR5A2* gene
P3	F: ATAGCTAGCGCAACACGAGGACAAGAG R: ATACTCGAGGGCAGTCCTTTGGTTTAG	56	1357	constructed pGL3-1373 vector of *NR5A2* gene
P4	F: AGAGCCTAAAACTAACCTTGGTC R: AGAAGCGGAGAGTTGGTGTG	55	575	SNP screening of *NR5A2* promoter
P5	F: CTGCCTGAAGGGCTCCAAAGTGCCAATCATGTCC R: CTTGCTGAGCCTGCGACCGTTCGTCTCTTCTTGT	56	705	Constructed pGL3-T vector of *NR5A2* gene
P6	F: AGGACCCTGGCACTTTGGAG R: TATTTCTTGGCATGGGCGTG	56	195	primers of *NR5A2* for real-time PCR
P7	F: AGCCTTCCTTCCTGGGCATGGA R: GGACAGCACCGTGTTGGCGTAGA	68	113	primers of β-actin for real-time PCR
P8	F: ATACTCGAGCACTGCCTTTCCATTTCTC R: ATATCTAGAGATGCTCCTGGGATACGA	55	2462	*mtf-1* overexpression
P9	F: 5’biotin- AGACGAACGGGCGCAGGCTCAGCAA R: 5’biotin- TTGCTGAGCCTGCGCCCGTTCGTCT			Bio-probe
P10	F: AGACGAACGTTTGCAGGCTCAGCAA R: TTGCTGAGCCTGCAAACGTTCGTC			Mut-probe
P11	F: AGACGAACGGGCGCAGGCTCAGCAA R: TTGCTGAGCCTGCGCCCGTTCGTCT			cold probe

Underline indicates restriction enzyme sites

*NR5A2* promoter sequences were screened for SNPs using a DNA pooling sequencing assay with primers P4 including core promoter region of *NR5A2* gene (**Table 1**). Five microliters of 100 ng/µl DNA was collected from five ewes and pooled together. PCR products were sequenced in both directions. SNPs were identified using Chromas v2.32 (Technelysium, Australia). SNPs of 193 ewes were genotyped with PCR-RFLP using Hin6I endonuclease (Takara, Japan) at 37°C for 3 h.

### Bioinformatic Analysis

The *NR5A2* promoter sequence was analyzed using DNASTAR (DNASTAR Inc., Madison, WI). The core promoter was predicted using Promoter 2.0 (Knudsen, 1999) and transcription binding sites in the promoter were predicted using JASPAR (http://jaspar.binf.ku.dk/).

### Construction of Plasmids

To identify the core promoter region, three *NR5A2* promoter deletion fragments (1: -281 to -17 bp; 2: -721 to -17 bp; 3: -1373 to -17 bp; these three fragments were only different in length but did not include deletions of different functional elements) were amplified and cloned in the NheI–XhoI (Takara, Japan) site of a pGL3-basic vector to create pGL3-281, pGL3-721, and pGL3-1373 products. The MTF1 CDS sequence of Hu sheep was amplified and cloned in the XhoI–XbaI (Takara, Japan) site of a *p*cDNA3.1(+) (Invitrogen, USA) vector to obtain the overexpression vector *p*cDNA3.1-MTF-1. Plasmids were extracted with an Endo-free Plasmid Mini Kit (Tiangen, China).

The -700GG promoter region containing an MTF1-binding site was amplified and cloned into pGL3-basic to obtain pGL3-G, and the -700TT promoter region was amplified by point mutation primer P5 (**Table 1**) using a point mutation kit (Takara, Japan) and cloned into pGL3-basic to obtain pGL3-T.

Point mutation amplification was followed according to the manufacturer’s instruction. In brief, firstly, a pair of primers (P5) was designed which was adjacent at 5′-ends and opposite-direction at 3′-ends to introduce mutation site (T). High-fidelity polymerase (*Pyrobest* DNA Polymerase) was used to PCR amplification, then DNA was purified, next was Blunting kination and ligation reaction to corresponding plasmids.

### Granulosa Cell Separation and Cell Culture

Granulosa cells of Hu sheep were separated from the ovaries following the methods described by Du et al. (2016), cultured in a T25 flask for 48 h, then subcultured in six-well plates at 5×10^6^ cells/dish for transfection to measure the gene expression. Granulosa cells and the 293T cell line (purchased from COBIOER, Shanghai, China) were cultured in Dulbecco’s modified Eagle’s medium (GIBCO, Thermo Fisher, USA) and supplemented with 10% fetal bovine serum (GIBCO, Thermo Fisher, USA) and 100 U/ml penicillin/streptomycin (GIBCO, Thermo Fisher, Shanghai, China). 293T cell lines were thawed and cultured under humidified air containing 5% CO_2_ at 37°C and subcultured in 24-well plates at 1×10^5^ cells/dish for transfection to measure the luciferase activity.

### Transfection and Dual-Luciferase Reporter Assay

Luciferase reporter plasmids were transfected into 293T cell lines using Lipofectamine 3000 (Invitrogen, USA) according to the manufacturer’s instructions. Briefly, 293T cells were seeded to be 70–90% confluent at 24-well plates for transfection. Then, Lipofectamine 3000 reagent diluted in Opti-MEM medium was added and mixed. A master mix of different constructs was prepared by diluting constructs in Opti-MEM medium, adding P300 reagent and mixing well. Diluted constructs were added to each tube of diluted Lipofectamine 3000 reagent (1:1 ratio) and incubated for 25 min. Finally, the constructs–lipid complex was added to cells. Forty-eight hours after transfection, the cells were harvested and treated for measuring the luciferase activity; all remaining steps were performed following the methods described by Wang et al. (2019).

### Competitive Electrophoretic Mobility Shift Assay (EMSA)

Nuclear extracts were prepared from ovine ovaries using a cytoplasmic and nuclear extraction kit (Viagene Biotech, China) according to the manufacturer’s instructions. Synthetic oligonucleotides 5′-labeled with biotin (Invitrogen, USA) were annealed to generate double-stranded oligonucleotides as probes (**Table 1**). The bio-probe is a wild-type probe labeled with biotin at the 5′ end; the cold probe, also called the competitive probe, is unlabeled and has the same sequence as that of labeled probes; the mut-probe is also unlabeled, and has the sequence as that of labeled probes, but the binding domain has been mutated with two bases. EMSAs were performed according to the instructions provided in the Competitive EMSA Kit (Viagene Biotech, Changzhou, China). Briefly, 3 µg of nuclear extract was incubated at 20°C for 20 min in the presence of 1.5 µl 10× binding buffer, 0.25 µl Poly (dI-dC) in a reaction volume of 14.5 µl, and then 0.5 µl of bio-probe was added, mixed well, and let the mixture sit at 20°C for 20 min. For competition experiments, unlabeled wild-type oligonucleotides (cold probe) and unlabeled mutant-type oligonucleotides (mut-probe) were added in a 100× molar excess prior to the addition of the biotinylated probe. Samples were electrophoresed through a 4% non-denaturing polyacrylamide gel in 0.5× Tris/Borate/EDTA at 4°C until the bromophenol blue reached the lower end of the gels (about 50–80 min), then electro-transferred and cross-linked by ultraviolet. Chemiluminescence imaging showed bands of DNA binding to transcription factors.

### Statistical Analyses

The allele frequencies, heterozygosity, and polymorphism information content were calculated using PopGene v1.31 software (http://www.ualberta.ca/∼fyeh/fyeh). Linkage analyses among different loci were performed with Haploview 4.2 (Barrett et al., 2005). The association analysis between different genotypes and the litter size of Hu sheep was referenced by Li et al. (2015). All results are reported as mean values ± SEM. Statistical analysis was performed with independent sample *t*-tests using SPSS 16.0 statistical software (SPSS Inc., USA).

## Results

### Identification of the Core Promoter Region of the Ovine *NR5A2* Gene

**Figure 1:**
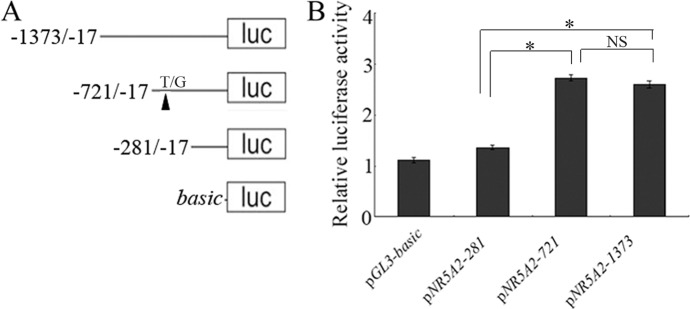
Identification of the core promoter in the ovine *NR5A2* gene. **(A)** Schematic diagram of the deletion constructs. The translation initiation site was defined as +1. **(B)** Luciferase assay. The deletion constructs were transfected into HEK293T cells. The results are expressed as the mean ± SEM (n = 3) in arbitrary units based on firefly luciferase activity normalized against *Renilla* luciferase activity. A *t*-test was conducted using SPSS 16.0 to detect the differences. The results are an average of three independent experiments performed in triplicate. *p < 0.05Q. NS: no significance.

To identify the core promoter region of *NR5A2* gene, three deletion plasmids, pGL3-281, pGL3-721, and pGL3-1373 (**Figure 1**), were transfected into 293T cells. The luciferase assay showed that the luciferase activity of plasmid pGL3-721 was significantly higher than that of pGL3-basic and pGL3-281 (*p* < 0.01). However, the difference in luciferase activity was not statistically significant between pGL3-721 and pGL3-1373 (*p* > 0.05), which revealed that the location of the core promoter region of the ovine *NR5A2* gene ranged from -721 to -281 bp (**Figure 1**). Additionally, many binding sites for transcription factors, such as C/EBPα, NF-Y, c-FOS, AP3, GATA2, SBF1, USF2, and MTF-1, were predicted to be present in this region (**Supplementary Figure 1**).

### SNP Identification in the Core Promoter Region and Correlation With Litter Size in Hu Sheep

SNPs in the *NR5A2* gene promoter region were explored by DNA pool sequencing, and a T/G mutation was identified at -700 nt in the core promoter region (**Supplementary Figure 2A**). Genotyping showed that GG, GT, and TT genotypes were found in 192 Hu ewes (**Supplementary Figure 2B**), with genotype frequencies of 0.104, 0.563, and 0.333, respectively. Association analysis showed that the litter sizes of Hu ewes at their second and average parities with genotype GG (2.20 ± 0.20 and 1.97 ± 0.06, respectively) were significantly higher than those of ewes with genotype TG (1.68 ± 0.10 and 1.74 ± 0.05, respectively; *p* < 0.05) and TT (1.67 ± 0.10 and 1.62 ± 0.06, respectively; *p* < 0.05). The litter size of Hu ewes at their third parity with genotype GG (2.10 ± 0.10) was significantly higher than that of ewes with genotype TT (1.56 ± 0.12; *p* < 0.05), and there was no significant difference between the litter sizes of ewes with genotypes GG (2.10 ± 0.10) and TG (1.85 ± 0.10; *p* > 0.05) (**Table 2**). These results indicate that the -700T/G polymorphism in the core promoter region of *NR5A2* gene was involved in regulating the litter size of Hu sheep.

**Table 2:** Average number of lambs and corresponding standard errors per parity of Hu sheep at G-700T.

genotye	number	1^st^ parity litter size	2^nd^ parity litter size	3^rd^ parity litter size	average litter size
GG	20	1.80 ± 0.13a	2.20 ± 0.20a	2.10 ± 0.10a	1.97 ± 0.06a
GT	108	1.63 ± 0.07a	1.68 ± 0.10b	1.85 ± 0.10ab	1.74 ± 0.05b
TT	64	1.56 ± 0.12a	1.67 ± 0.10bc	1.56 ± 0.12c	1.62 ± 0.06bc

values with different superscripts within the same column in particular locus differ significantly at P < 0.05.

### The -700T/G Polymorphism Affected Promoter Activity of the *NR5A2* Gene

**Figure 2:**
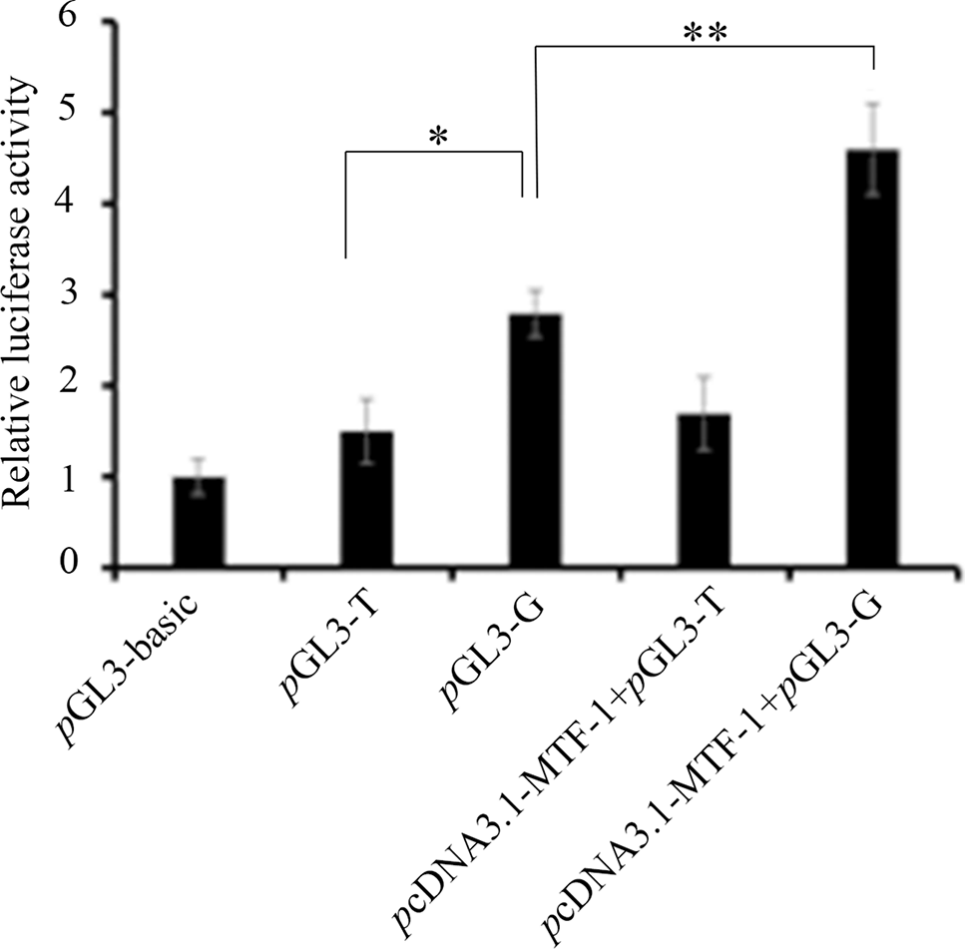
The -700T/G mutation regulates NR5A2 promoter activity. Constructs of pGL3-G (containing MTF-1 binding site) and pGL3-T (no MTF-1 binding site) were transfected or co-transfected with pcDNA3.1-MTF-1 into HEK293T cells, and luciferase activity was detected using a Dual-Luciferase Reporter Assay System. Results are expressed as the mean ± SEM (n = 3). *p < 0.05; **p < 0.01.

To investigate whether the -700T/G polymorphism affected promoter activity of the *NR5A2* gene, constructs of pGL3-T and pGL3-G were transfected into 293T cells, and the results showed that luciferase activity of plasmid pGL3-G was significantly higher than that of plasmid pGL3-T in 293T cells (*p* < 0.05) (**Figure 2**).

### The -700T/G Polymorphism Adds a Novel Transcription Factor Binding Site

**Figure 3:**
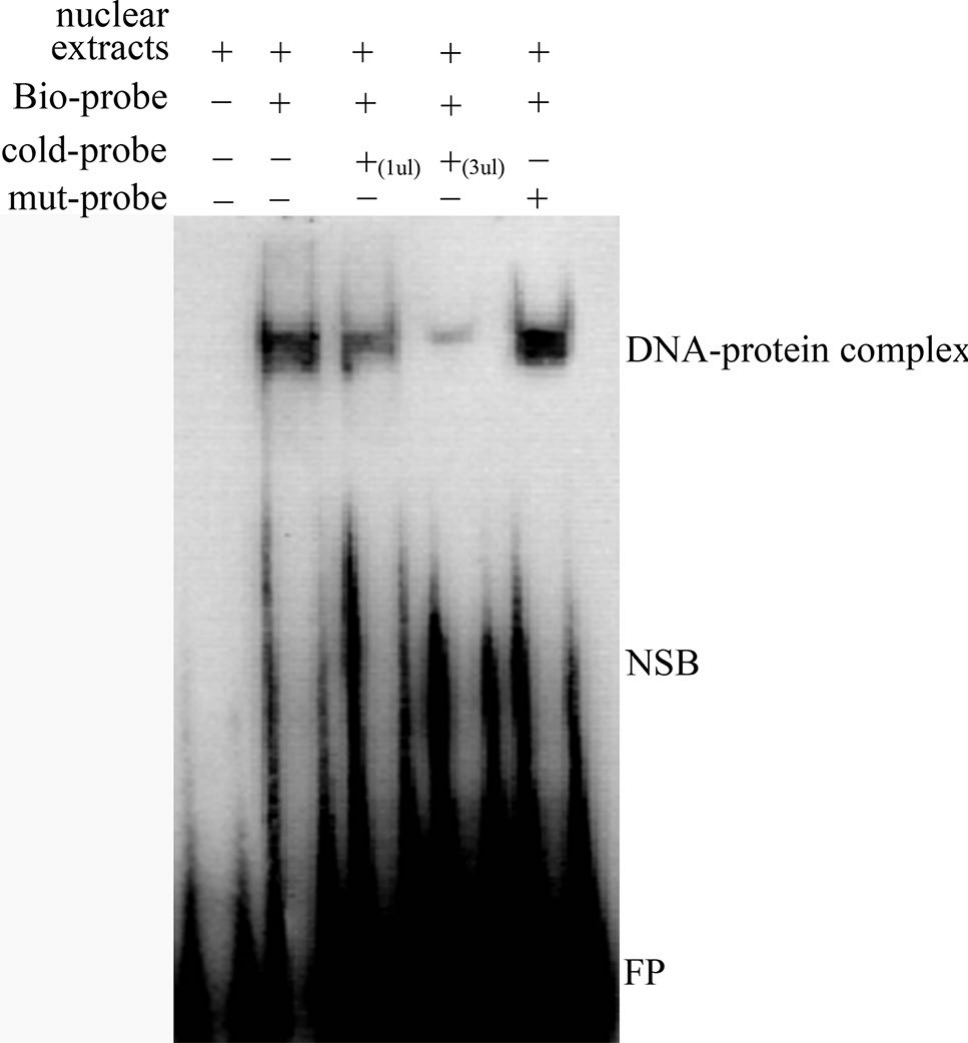
Competitive EMSA assay confirms the specific binding between MTF-1 and -700GG of the NR5A2 promoter region. Probes in this assay containing the NR5A2 -700GG site with or without biotin labeling were called bio-probes or cold probes, and probes containing the NR5A2 -700TT site without biotin labeling were called mut-probes. These probes were incubated with ovary nuclear extracts in different combinations, and DNA–protein complexes were visualized by autoradiography. FP, free probes; NSB, non-specific binding; DNA–protein complex: DNA and transcription factor MTF-1 binding complex.

We predicted that the -700T/G polymorphism would create a novel binding site for transcription factor MTF-1. A competitive EMSA showed that when nuclear extracts of ewes’ ovaries were incubated with a bio-probe, there was a significant DNA–protein complex, and when nuclear extracts were incubated with a bio-probe and mut-probe at the same time, the DNA–protein complex was still present. However, when nuclear extracts were incubated with a bio-probe and cold probe at the same time, DNA–protein complex significantly decreased with the cold-probe concentration increased. These results suggest that MTF-1 binds specifically to sequences with -700GG (**Figure 3**).

### MTF-1 Was Involved in Regulating *NR5A2* Gene Expression

**Figure 4:**
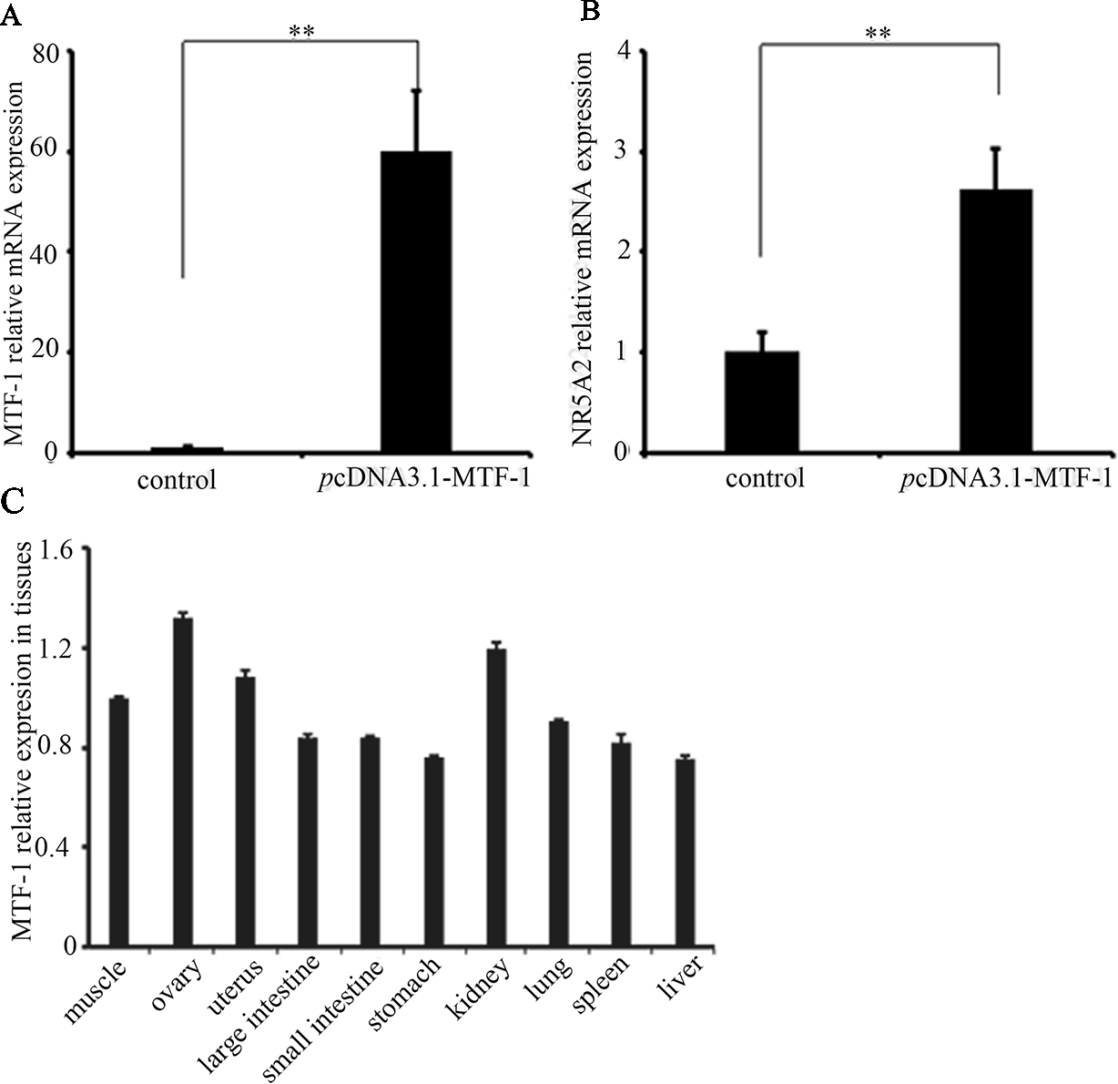
MTF-1 involved in regulating *NR5A2* gene expression in ovine ovarian granulosa cells. **(A)** MTF-1 gene expression after transfection in pcDNA3.1-MTF-1 in ovine ovarian granulosa cells. **(B)** Overexpression of MTF-1 increases the *NR5A2* gene expression in ovine ovarian granulosa cells. **(C)** MTF-1 expression profiles in ovine tissues. **p < 0.01.

To determine whether MTF-1 was involved in regulating *NR5A2* gene expression, the plasmid *p*cDNA3.1-MTF-1 was transfected in sheep granulosa cells. The qRT-PCR results showed that the MTF-1 expression levels were higher in sheep granulosa cells transfected with plasmid pcDNA3.1-MTF-1 (*p* < 0.01) (**Figure 4**), and expression levels of the *NR5A2* gene increased after pcDNA3.1-MTF-1 transfection (**Figure 4**). Additionally, qRT-PCR results showed that MTF-1 was widely expressed in Hu sheep tissues, particularly in the ovary and uterus (**Figure 4**). These data suggest that MTF-1 was involved in regulating *NR5A2* gene expression in the ovarian granulosa cells of Hu sheep.

### -700T/G Polymorphism Affected MTF-1 Regulation of NR5A2 Promoter Activity

*p*cDNA3.1-MTF-1 was co-transfected with pGL3-T or pGL3-G into 293T cells. The promoter activity assay showed that the overexpression of MTF-1 significantly enhanced the luciferase activity of the -700G promoter genotype (*p* < 0.01), but the -700T promoter genotype did not have a significant effect on luciferase activity (*p* > 0.05) (**Figure 2**).

## Discussion

NR5A2 is a transcription factor involved in regulating metabolism and hormone synthesis (Cobo-Vuilleumier et al., 2018; Jiang et al., 2018). Studies have shown that NR5A2 is essential for pregnancy and reproduction in female animals (Brosens et al., 2013; Zhang et al., 2013; Guo et al., 2016; Kaluarachchi et al., 2016). A previous study showed that the expression level of *NR5A2* was associated with ovulation and litter size in Hu sheep (Li et al., 2015). In this study, we focused on the 5′ regulatory region of NR5A2 and determined that its core promoter region ranged from -721 to -182 nt. A -700T/G polymorphism was detected at the core promoter region of the *NR5A2* gene. Polymorphisms of *NR5A2* are associated with preterm birth in humans (Kaluarachchi et al., 2016), and it has been shown that T40C and T1419C at the NR5A2 CDS region are associated with litter size in Hu sheep (Li et al., 2015). In this study, we found that -700T/G located in the core promoter of NR5A2 was significantly associated with litter size in Hu sheep, with ewes with genotype GG having a significantly greater litter size at the second and average parities than those with genotypes TG (*p* < 0.05) and TT (*p* < 0.05). Moreover, ewes with genotype GG had a significantly greater litter size at the third parity than those with genotype TT (*p* < 0.05). By combining all three polymorphisms (-700T/G, T40C, and T1419C) of the *NR5A2* gene, we found that ewes with the GG/TC/TC haplotype have higher litter sizes in the second and third parities than those with other haplotypes (**Supplementary Table 1**), suggesting that the *NR5A2* gene could be used as a candidate molecular marker for breeding high-fertility sheep.

We found that the T/G polymorphism changed the promoter activity of the *NR5A2* gene, suggesting that this mutation was probably involved in regulating *NR5A2* gene expression, affecting the litter size of sheep as a result. Further analysis showed that -700G created a transcription factor MTF-1 binding site and MTF-1 was involved in regulating NR5A2 promoter transcription activity, increasing the *NR5A2* gene expression in granulosa cells of sheep. Xu et al. (2018) found that polymorphisms of the *HOMER1* gene are associated with piglet splay leg syndrome and the G allele of rs325197091 (A > G) may create a new binding site of transcription factor ARNT, which could enhance *HOMER1* promoter activity and expression. Promoter haplotypes of the *ABCB1* gene encoding the P-glycoprotein differentially affects its promoter activity by altering the transcription factor binding (Speidel et al., 2018).

**Figure 5:**
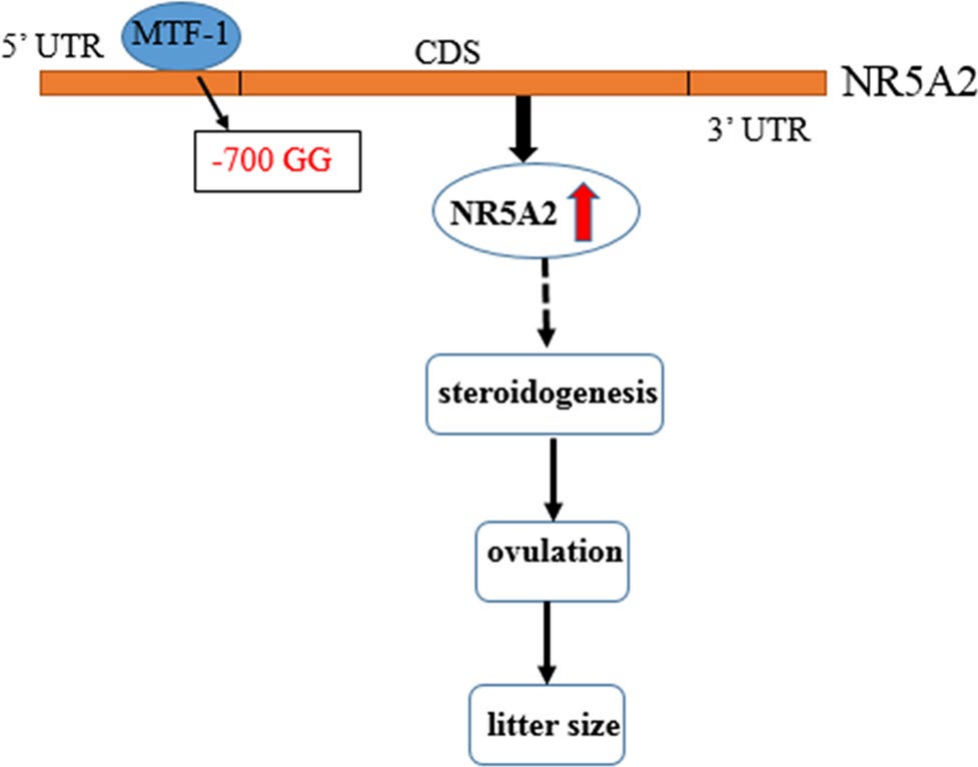
Flow diagram of the -700T/G mutation of *NR5A2* regulating ovine reproduction through *MTF-1*. -700T/G polymorphism of NR5A2 gene core promoter creates a novel transcription factor MTF-1 binding site, upregulates the transcription activity and mRNA expression of NR5A2 gene in granulosa cells, and then increases the litter size of Hu sheep probably due to regulating steroidogenesis of NR5A2 and further affecting ovulation and litter size.

MTF-1, a six-zinc-finger protein of the Cys2His2 family, binds directly and specifically to metal response elements, and regulates metal-induced and basal MT expression (Heuchel et al., 1994; Radtke et al., 1995). As a transcription activator, MTF-1 plays different roles and is involved in metal and oxidative stress (Dalton et al., 1996; Ben Mimouna et al., 2018) and embryological development (Hogstrand et al., 2008). Nishimoto et al. (2009) found that MTF-1, as a transcription activator, binds with the promoter region of PIGF, increases its expression, and is involved in the hypoxia-dependent regulation of PIGF in trophoblast-derived cells. MTF-1 could also be a transcription factor that binds to FSH, CYP19A2, and 20b-HSD, and is also involved in regulating steroidogenesis synthesis in zebrafish (Urbatzka et al., 2012). In this study, we found that MTF-1 is a transcription activator that can bind with an NR5A2 -700GG type promoter, increase its promoter activity and expression level in granulosa cells, and further regulate the ovulation and litter size of Hu sheep (**Figure 5**).

## Data Availability Statement

The raw data supporting the conclusions of this manuscript will be made available by the authors, without undue reservation, to any qualified researcher.

## Ethics Statement

All experiments were performed in accordance with the protocol approved by the Committee on Ethics of Animal Experimentation from the Jiangsu Academy of Agricultural Sciences (No. 63 of Jiangsu Academy of Agricultural Sciences, approved on 8 July 2014).

## Author Contributions

YL is responsible for the design and the writing of the whole article. JZa is responsible for SNP screening. YQ is responsible for samples collection. CM is responsible for real-time PCR. HW is responsible for cell assay. SC is responsible for EMSA assay. JZo is responsible for the modification of the whole article.

## Funding

The funding is from the National Natural Science Foundation of China, and the number is 31501934. The title of this funding is the Molecular Mechanisms of -700T/G Mutation Regulating the Transcription of NR5A2 and High Fecundity in Hu Sheep. This paper is completely consistent with this funding, and this funding includes the publication fees. Another funding is Agricultural Science and Technology Innovation of Jiangsu Province, the number is CX(2018)3004.

## Conflict of Interest

The authors declare that the research was conducted in the absence of any commercial or financial relationships that could be construed as a potential conflict of interest.

## Supplementary Material

Supplementary Figure 1 Characterization of the NR5A2 promoter region in Hu sheep.

Supplementary Figure 2 SNP identification and genotyping of the -700T/G mutation in the ovine NR5A2 gene.

Supplementary Table 1 The association between different haplotype and litter sizes in Hu sheep.
