# Supplementary figures and images for: A T > G Mutation in the NR5A2 Gene Is Associated With Litter Size in Hu Sheep Through Upregulation of Promoter Activity by Transcription Factor MTF-1
Source: Front Genet. 2019 Oct 25;10:1011. doi: 10.3389/fgene.2019.01011 (PMC6824215; doi:10.3389/fgene.2019.01011)

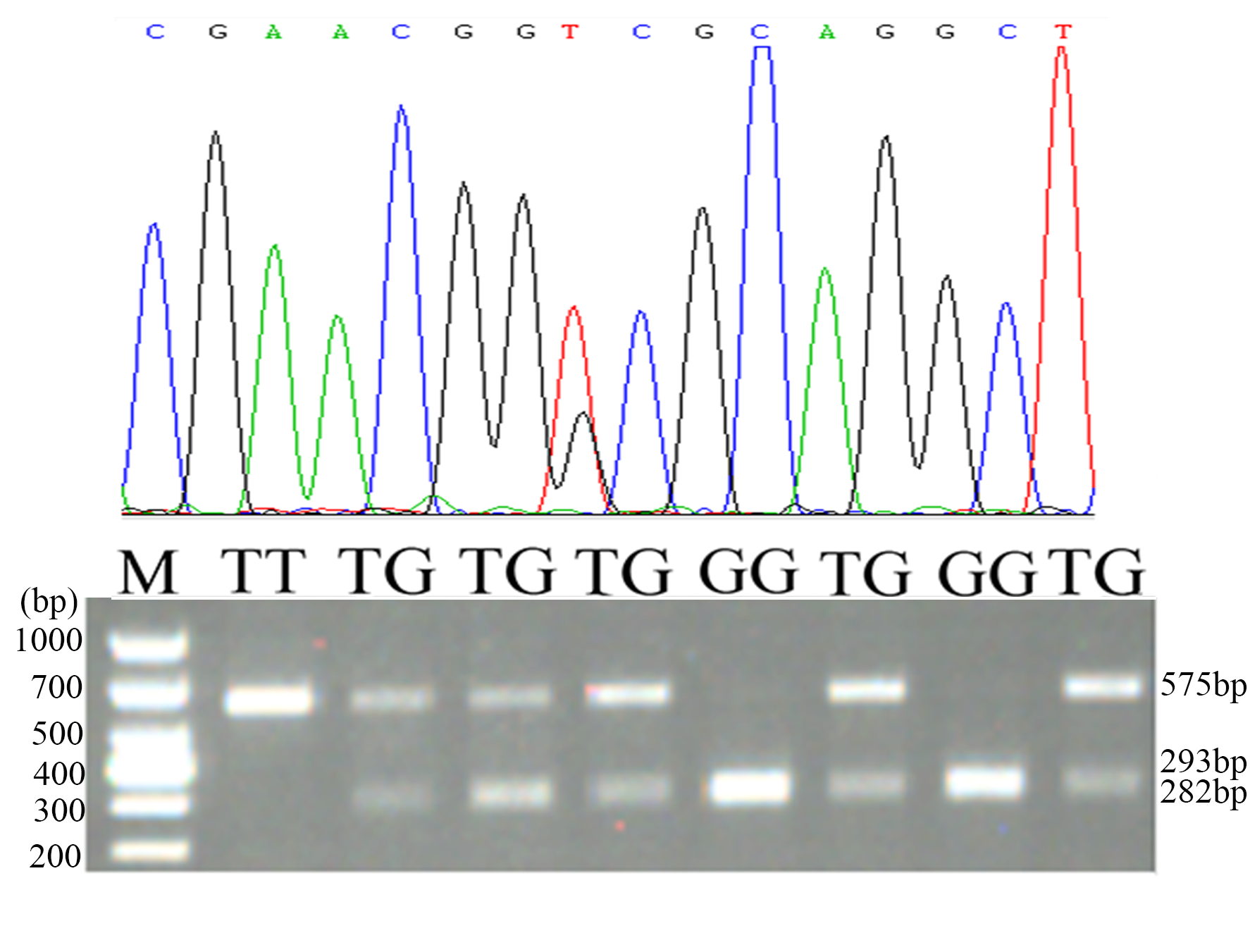

Supplement: Supplementary Figure 1 — Characterization of the NR5A2 promoter region in Hu sheep. [file Image_1.tif]

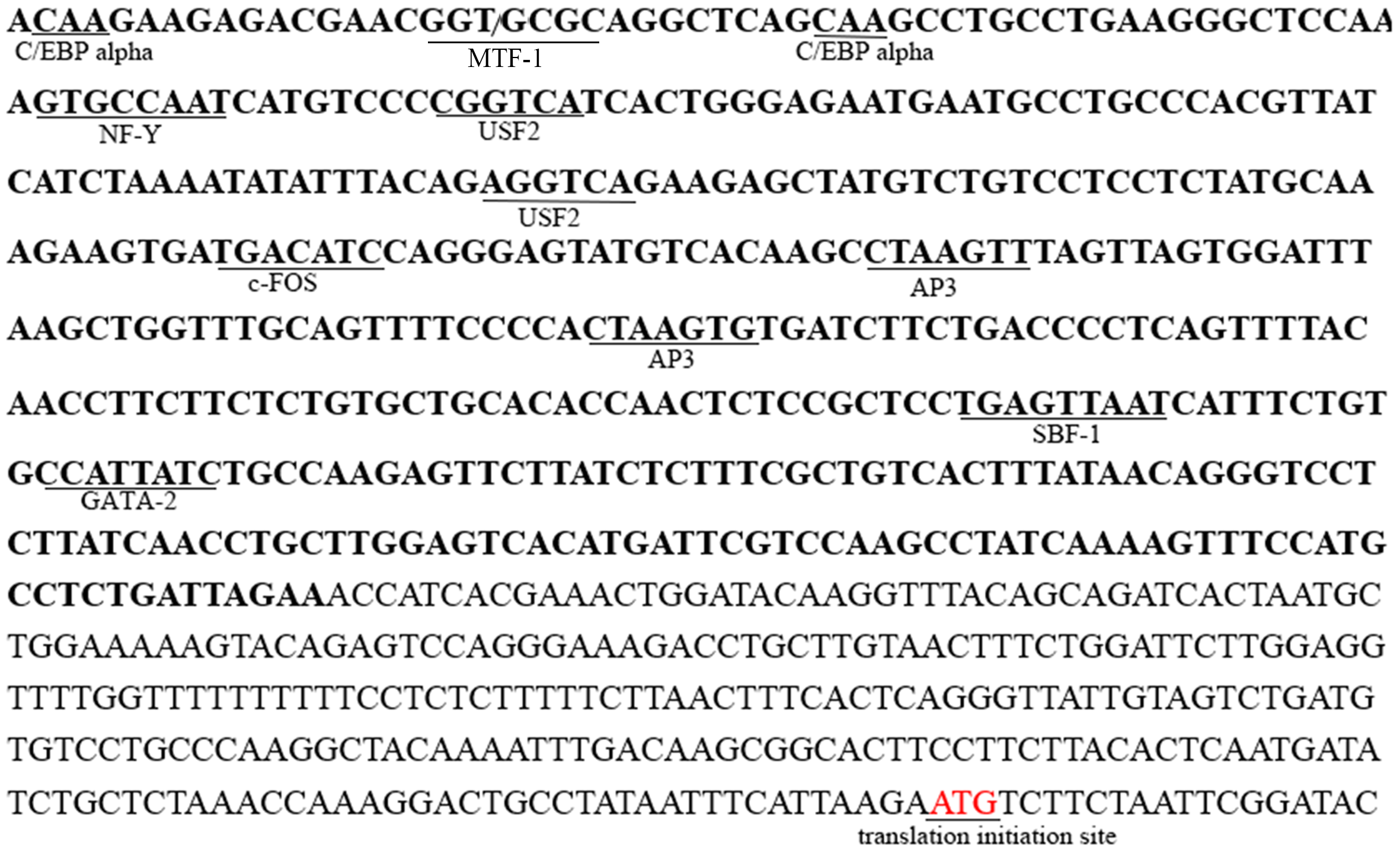

Supplement: Supplementary Figure 2 — SNP identification and genotyping of the -700T/G mutation in the ovine NR5A2 gene. [file Image_2.tif]
