# Supplementary material for: A T > G Mutation in the NR5A2 Gene Is Associated With Litter Size in Hu Sheep Through Upregulation of Promoter Activity by Transcription Factor MTF-1
Source: Front Genet. 2019 Oct 25;10:1011. doi: 10.3389/fgene.2019.01011 (PMC6824215; doi:10.3389/fgene.2019.01011)
Supplement: Supplementary Table 1 — The association between different haplotype and litter sizes in Hu sheep. [file Table_1.docx]

Supplementary table 1 the association between different haplotype and litter sizes in Hu sheep

| haplotype | The first parity (number) | The second parity (number) | The third parity (number) |
| --- | --- | --- | --- |
| GT/TC/TC | 1.66±0.58 (84) | 1.75±0.53a (64) | 1.76±0.53a (30) |
| TT/TC/TC | 1.61±0.59 (29) | 1.8±0.54ab (22) | 1.27±0.45cD (16) |
| TT/TT/TC | 1.60±0.49 (15) | 1.40±0.49ab (8) | 1.60±0.49abc (8) |
| GT/TC/TT | 1.50±0.50 (9) | 1.75±0.43ab (6) | 2.0±0.82abc (5) |
| GG/CC/TC | 1.83±0.69 (9) | 1.75±0.83ab (6) |  |
| GT/CC/TC | 1.57±0.49 (12) | 1.60±0.80ab (8) |  |
| TC/TT/GT | 1.80±0.40 (7) | 1.25±0.43c (6) |  |
| GG/TC/TC | 1.80±0.40 (7) | 2.2±0.75ad (7) | 2.0±0.00abE (7) |

Different lowercase letters represent significant differences (p<0.05), different uppercase letters represent extremely significant difference (p<0.01).

For the validity of the correlation analysis, the number of ewes less than 5 is not listed in this table.
